# 1HNMR-based metabolomic profile of rats with experimental acute pancreatitis

**DOI:** 10.1186/1471-230X-14-115

**Published:** 2014-06-30

**Authors:** Juan Li, Xian-lin Zhao, Yi-xia Liu, Xiao-hang Peng, Shi-feng Zhu, Hui Guo, Yi-Ling Liu, Mei-hua Wan, Wen-fu Tang

**Affiliations:** 1 Department of Integrative Medicine, West China Hospital, Sichuan University, Chengdu 610041, Sichuan Province, China; 2 China Tibetology Research Center, 100101 Beijing, China

**Keywords:** Metabolomics, Acute pancreatitis, 1HNMR, PCA analysis, PLS-DA analysis

## Abstract

**Background:**

Acute pancreatitis (AP) is a common inflammatory disease of the pancreas accompanied by serious metabolic disturbances. Nevertheless, the specific metabolic process of this disease is still unclear. Characterization of the metabolome may help identify biomarkers for AP. To identify potential biomarkers, this study therefore investigated the 1H-nuclear magnetic resonance (NMR)-based metabolomic profile of AP.

**Methods:**

Fourteen male adult Sprague–Dawley rats were randomized into two groups: the AP group, in which AP was induced by retrograde ductal infusion of 3.5% sodium taurocholate; and the sham operation group (SO), in which rats were infused with 0.9% saline. Blood samples were obtained 12 hours later and a 600 MHz superconducting NMR spectrometer was used to detect plasma metabolites. Principal components analysis (PCA) and partial least squares-discriminant analysis after orthogonal signal correction (OSC-PLS-DA) were used to analyze both longitudinal Eddy-delay (LED) and Carr–Purcell–Meiboom–Gill (CPMG) spectra.

**Results:**

Differences in plasma metabolites between the two groups were detected by PCA and PLS-DA of 1HNMR spectra. Compared with the SO group, plasma levels of lactate (δ 1.3, 1.34, 4.1), valine (δ 0.98, 1.02), succinic acid (δ 2.38), 3-hydroxybutyric acid (3-HB, δ 1.18), high density lipoprotein (HDL, δ 0.8), and unsaturated fatty acid (UFA, δ 2.78, 5.3) were elevated in the AP group, while levels of glycerol (δ 3.58, 3.66), choline (δ 3.22), trimethylamine oxide (TMAO, δ 3.26), glucose (δ 3–4), glycine (δ 3.54), very low density lipoprotein (VLDL, δ 1.34) and phosphatidylcholine (Ptd, δ 2.78) were decreased.

**Conclusions:**

AP has a characteristic metabolic profile. Lactate, valine, succinic acid, 3-HB, HDL, UFA, glycerol, choline, TMAO, glucose, glycine, VLDL, and Ptd may be potential biomarkers of early stage AP.

## Background

Acute pancreatitis (AP) is a common inflammatory disease of the pancreas caused by premature activation of pancreatic enzymes. Although AP is self-limiting in 80% of patients, it can be life-threatening in the 20% who develop systemic complications, such as multiple organ failure [1]. In addition to a high mortality rate, ranging from 3–15% [2], the rate of hospitalization for AP continues to rise annually [3]. In the United States, the overall hospitalization rate has doubled over the past 20 years [4], and in the Netherlands there was a 75% increase from 1992 to 2004 [5]. A recent study suggested a 3.1% annual increase in the incidence of AP [6] in England, and a meta-analysis from 18 European countries showed that the incidence of biliary pancreatitis has increased linearly and that the mortality rate increases with age [7]. Similar findings were reported in a study investigating the epidemiology of AP in the North Adriatic region of Croatia [8]. A population-based study from Taiwan reported that the proportion of severe cases has increased in recent years, although the overall incidence of AP remained constant [9]. Moreover, throughout the world, AP poses a heavy financial burden on the health care system. Due to unclear pathogenesis, the main treatments of AP are still supportive and symptomatic therapies, although early diagnosis and intervention may mitigate illness and reduce complications and length of hospitalization [10-13]. Metabolic disturbances, such as hyperglycemia and hyperlipidemia, are always present in the early stages of AP. However, the specific metabolic processes associated with this disease remain unclear.

Metabolomics is a modern approach to the study of biological samples, and can provide detailed information on the metabolic changes taking place in an organism in various pathophysiologic states. ^1^H NMR spectroscopy-based approaches are used for high-throughput research on biological samples [14-16]. Moreover, being non-invasive and providing an impartial profile of all metabolites, ^1^H NMR-based metabolomics is widely applied in many areas related to biology and medicine, such as the exploitation of new drugs, disease diagnosis, toxicology, pharmaceutical effects, microorganism metabolomics, and identification of biomarkers [17-21]. Detection of characteristic metabolite changes in AP may increase our understanding of the pathophysiology of this disease, allow early diagnosis, and identify potential therapeutic targets. To identify potential biomarkers of AP, we analyzed the metabolic differences between rats with AP and healthy rats.

## Methods

### Ethics statement

This prospective, randomized controlled trial was performed at the ethnopharmacology laboratory of West China Hospital. The study protocol was approved by the Ethics Committee for Animal Experiments of Sichuan University. All rats were handled according to University Guidelines and the Animal Care Committee Guidelines of West China Hospital. All operations were performed under chloral hydrate anesthesia, and all efforts were made to minimize suffering.

### Chemicals

Deuterium oxide (D_2_O, 99.9%) was purchased from Cambridge Isotope Laboratories, Inc (Tewksbury, MA, USA). Trimethylsilyl-propionate-2, 2, 3, 3-d_4_ acid, sodium salt (TSP) was purchased from Merck (Darmstadt, Germany). Sodium taurocholate was provided by Sigma Chemical (St. Louis, MO, USA). Chloral hydrate was provided by Ke Long chemical reagent works, China.

### Equipment

An INOVA 600 MHz NMR spectrometer equipped with a triple-resonance probe and a z-axis pulsed field gradient was obtained from Varian Unity (Varian, Inc. USA), a microcentrifuge from Eppendorf MiniSpin Plus, Germany, and a two-channel micro-injection pump from Kd Scientific Company, USA.

### Animal model of acute pancreatitis

Healthy male adult Sprague–Dawley rats (224 ± 21 g, 200–250 g b/w), were maintained in air-conditioned animal quarters at 22 ± 2°C with a relative humidity of 65% ± 10%. They were acclimated for 1 week before the experiment with special feed and tap water. The animal experiments were carried out in the Laboratory of Ethnopharmacology at West China hospital, Sichuan University.

Fourteen 2-month-old (55–62 days old) rats obtained from the experimental animal center of Sichuan University were numbered and randomized into two groups of seven rats each, an AP group and a sham operation (SO) group. Animals were fasted for 12 h, and given free access to tap water prior to experiments. All animals were anesthetized by intraperitoneal injection of 3 ml/kg 10% chloral hydrate. The hepatic duct was closed with a small bulldog clamp, and the biliopancreatic duct was cannulated through the offside of the front opening of the duodenum papilla. Rats in the AP group underwent retrograde pancreaticobiliary duct injection with 1 ml/kg 3.5% sodium taurocholate using an infusion pump, whereas rats in the SO group were similarly injected with 1 ml/kg 0.9% saline [22]. Twelve hours later, heparinized blood samples were obtained from the angular vein and centrifuged for 15 minutes at 3000 rpm. Plasma samples were collected and stored at −80°C until use. After the experiment, the rats were sacrificed and cremated.

### Sample preparation and NMR data acquisition

Plasma samples (150 μl) were mixed with 100 μl 1 mg/ml TSP in deuterium oxide D_2_O and 350 μl D2O, and centrifuged at 14000 rpm for 10 min. A 550 μl aliquot of supernatant was transferred into 5 mm NMR tubes, and NMR spectra were recorded at 26°C using an INOVA 600 MHz NMR spectrometer equipped with a triple-resonance probe and a z-axis pulsed field gradient. Information about micromolecular metabolites was acquired using a CPMG pulse sequence [−RD–90°–(r–180°–r)_n_–ACQ] during a relaxation delay of 2 s. Sixty-four free induction decays (FID) were collected into 64 k data points with a spectral width of 8000 Hz and acquisition time of 4 s. Information about macromolecular metabolites was collected by LED pulse sequence (−RD-90°-G1-180°-G1-90°-T-90°-G1-180°-G1-90°-ACQ) during a relaxation delay of 2 s. Sixty-four FID were collected into 64 k data points with a spectral width of 8000 Hz, an acquisition time of 4 s and a diffusion time of 100 min. FID was zero-filled and multiplied by an exponential line-broadening function of 1 Hz (for CPMG) and 3 Hz (for LED) prior to Fourier transformation.

### NMR data processing and analysis

After Fourier transformation, the data were phased and subjected to baseline correction. For CPMG data, spectra in the region of δ 0.4–4.4 were subdivided into integrated regions of 0.04 PPM width. LED data were subdivided into integrated regions of 0.04 PPM width corresponding to the region of δ 0–6.0, and the spectra in the δ 4.6–5.0 range were excluded from data reduction. Each data point was normalized to the sum of its row to compensate for any variation in total sample volumes. The obtained data were exported to Excel and subjected to multivariate analyses as variables for the multivariate pattern recognition analysis using Soft Independent Modeling of Class Analogy (SIMCA-P) software package (v10.04, Umetrics, Umeå, Sweden). Principal components analysis (PCA) was performed after data were pretreated by mean centering and Pareto scaling. For samples with obvious internal individual differences, partial least squares (PLS) and discriminant analysis (DA) were conducted after data processing based on orthogonal signal correction (OSC). These two approaches were performed to differentiate between sample groups. Score plots were used to visualize the separation of the groups, while the loading plots were used to determine which spectral variables significantly contributed to the separation of the samples on the score plots.

## Results

### 1H-NMR spectra (CPMG) and pattern recognition analysis of plasma samples

**Figure 1:**
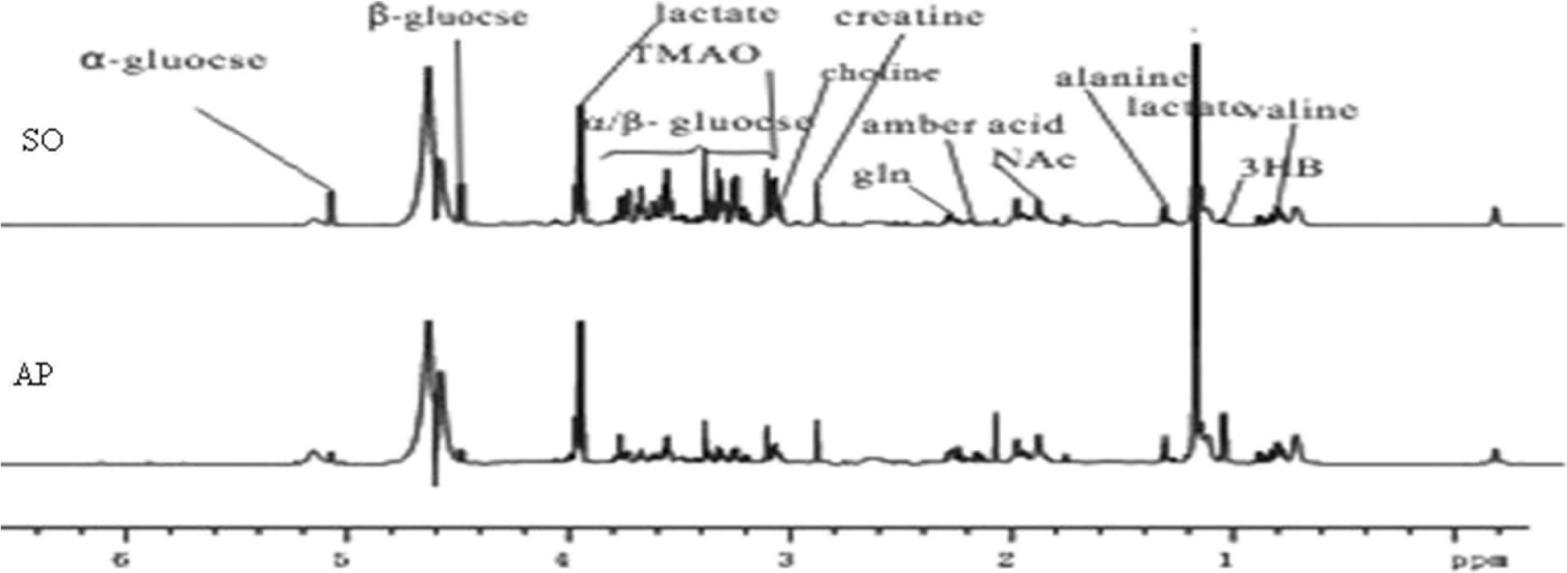
**Plasma 1H-NMR spectrogram of CPMG.** Sham operation group (SO) and acute pancreatitis group (AP).

**Figure 2:**
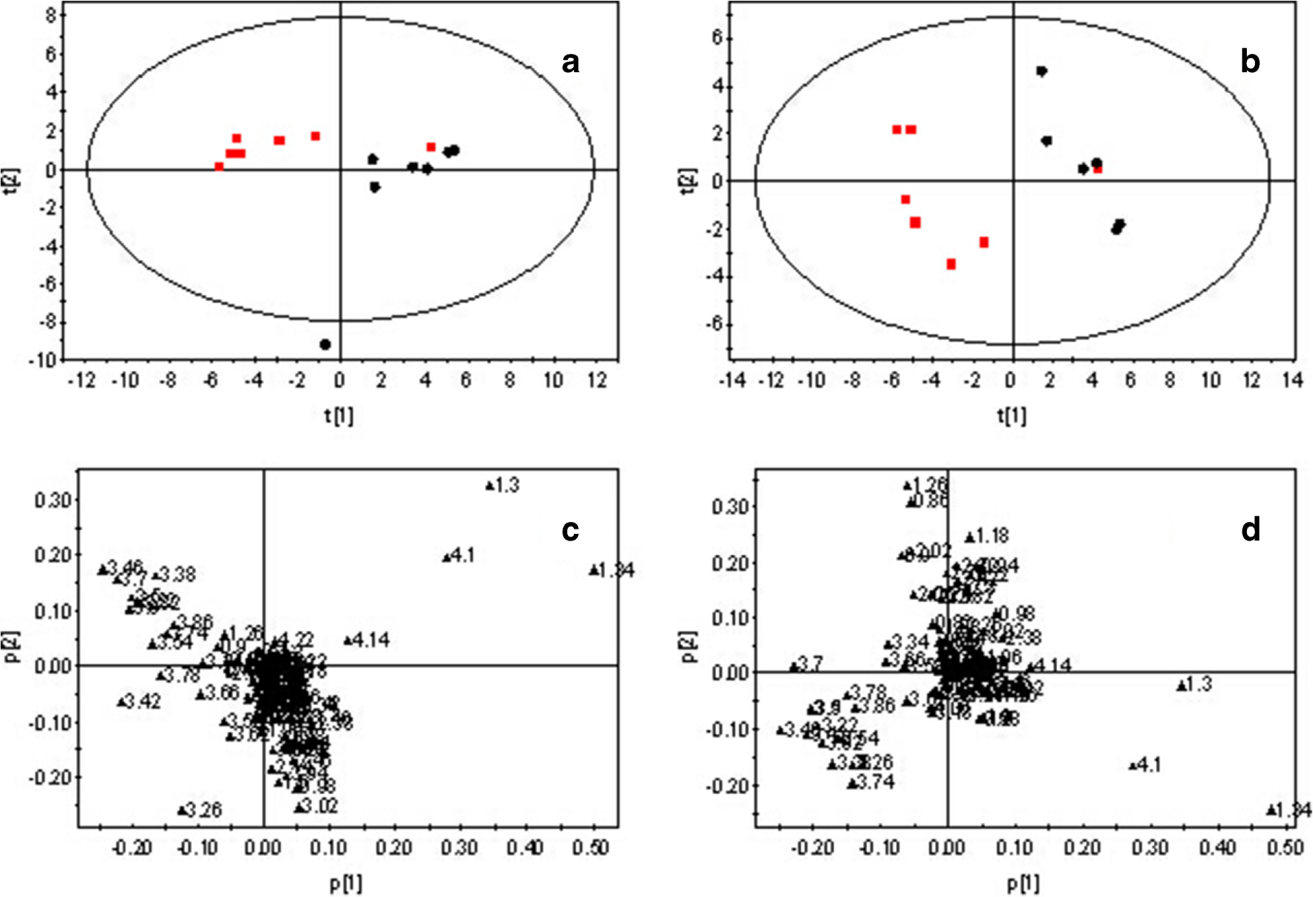
**1H-NMR (CPMG) spectra of plasma samples.** Samples from SO (red square symbol) and AP (●) groups. **(a)** Score plot of PCA analysis shows cluster distribution of the two groups but without distinct separation, with the point y10 located outside the confidence interval. **(b)** Score plot of PCA analysis after excluding the AP10 point. **(c)** Loading plot of PCA analysis. **(d)** Loading plot of PCA analysis after excluding the AP10 point. R2X (cum) =84.9%; Q2 (cum) =75.9%.

A comparison of the 1H-NMR spectra of plasma samples taken from rats in the AP and SO groups showed obvious differences in the levels of small molecule metabolites (Figure 1). To identify these differences in metabolic profiles, the 1H-NMR spectra were segmented and subjected to PCA. The scores plot showed a cluster distribution of the two groups without distinct separation, with the point AP10 located outside the confidence interval (Figure 2a). The clusters became more obviously distributed after excluding the AP10 point (Figure 2b). With the loading plot (Figure 2d), a sequence of metabolic alterations was detected in plasma samples from rats with AP: (1) increased levels of lactate, valine, succinic acid, and 3-hydroxybutyric acid (3-HB); and (2) decreased levels of glycerol, choline, trimethylamine oxide (TMAO), glucose, and glycine (Table 1). These altered metabolites may be potential biomarkers of AP.

**Table 1:** Levels of small molecule metabolites in the AP and SO groups

**Chemical shift (ppm)**	**Name of compound**	**Acute pancreatitis group (AP)**
**0.98,1.02**	**Valine**	↑
**1.3,1.34,4.1**	**Lactate**	↑
**3.58,3.66**	**Glycerol**	↓
**2.38**	**succinic acid**	↑
**1.18**	**3-HB**	↑
**3.22**	**Choline**	↓
**3.26**	**TMAO**	↓
**3 ~ 4**	**Glucose**	↓
**3.54**	**Glycine**	↓

↑: elevated; ↓: reduced; 3-HB: 3-hydroxybutyric acid; TMAO: trimethylamine oxide. All of the results were compared with sham operation (SO) group.

### 1H-NMR spectra (LED) and pattern recognition analysis of plasma samples

**Figure 3:**
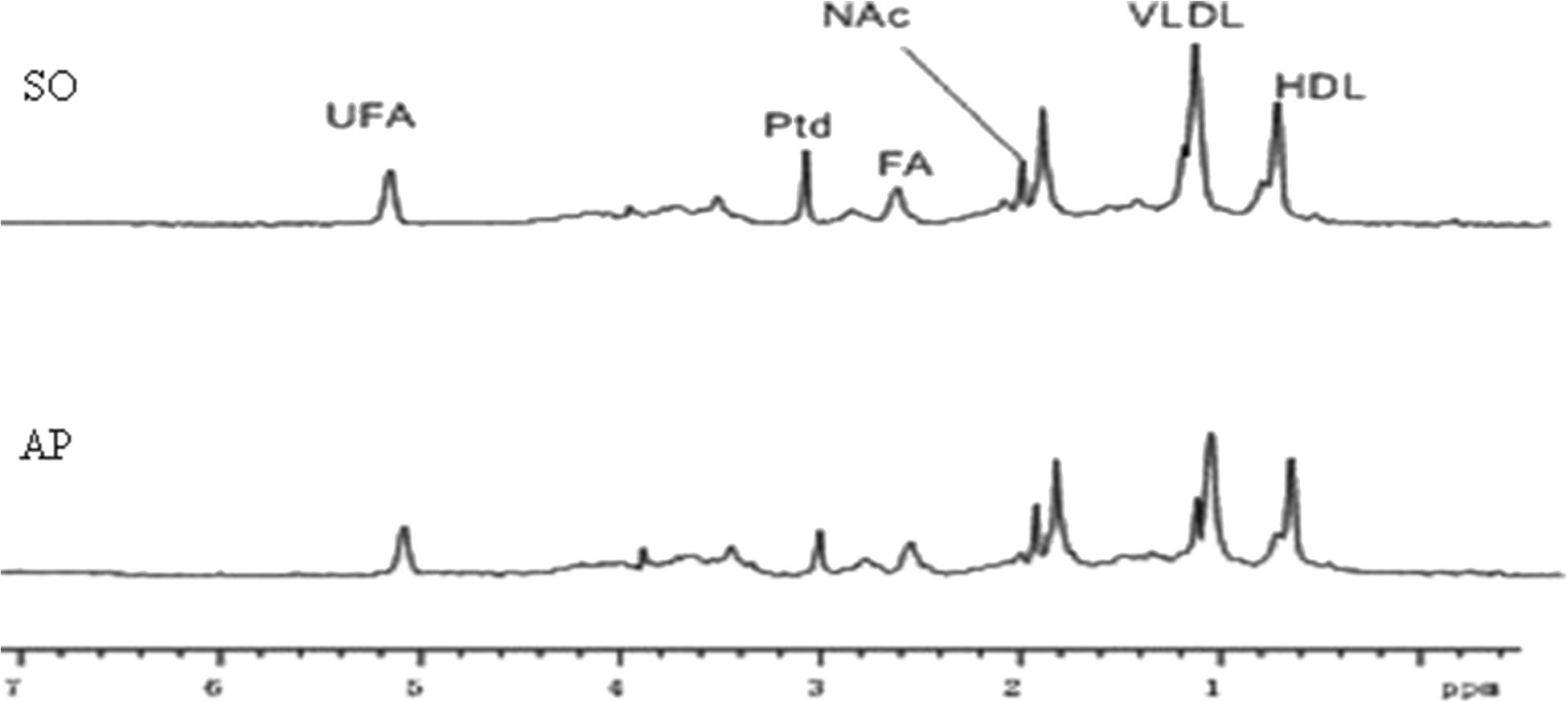
**Plasma 1H-NMR spectrogram of LED.** Sham operation group (SO) and acute pancreatitis group (AP).

**Figure 4:**
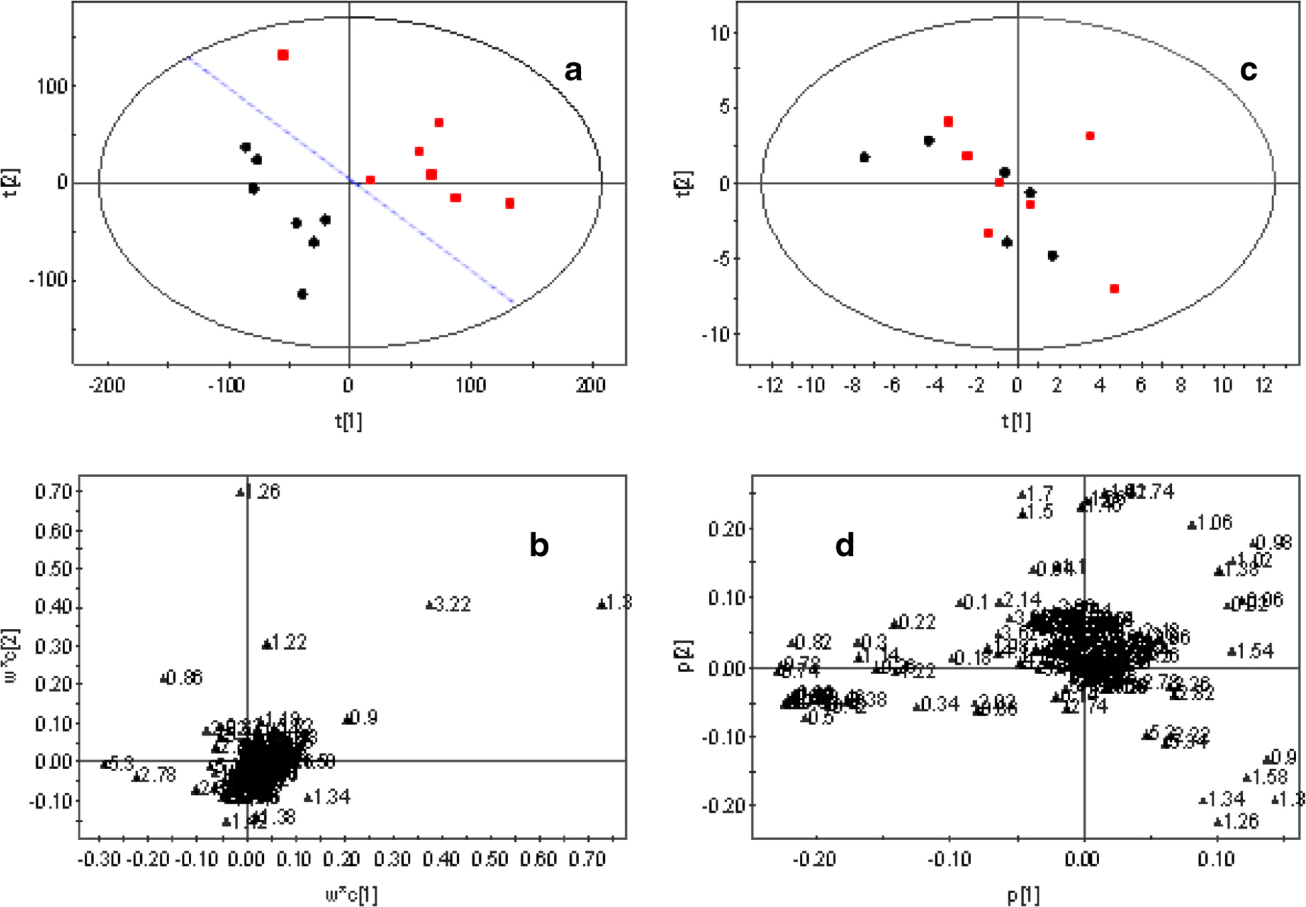
**1H-NMR (LED) spectra of plasma samples.** Samples from SO (red square symbol) and AP (●) groups. **(a)** Score plot of OSC-PLS-DA. **(b)** Loading plot of OSC-PLS-DA. R2X (cum) = 70.2%; R2Y (cum) = 87.1%; Q2 (cum) = 72.4%. **(c)** Score plot of PCA analysis. **(d)** Loading plot of PCA analysis.

Similar to the analysis of small molecule metabolites, the levels of macromolecule lipid metabolites differed markedly between the AP and SO groups, as shown by the 1H-NMR (LED) spectra of their plasma samples (Figure 3). To illustrate the differences in metabolic profiles required OSC-PLS (ctr) analysis (Figure 4a,b) was conducted because PCA analysis was not able to detect differences between these two groups (Figure 4c,d). The score plot showed distinct separation between the two groups (Figure 4a), with the loading plot (Figure 4b) showing increases in high density lipoproteins (HDL, 0.86) and unsaturated fatty acids (UFA, 2.78, 5.3), and decreases in very low density lipoprotein [(VLDL, 1.34), (VLDL/LDL, 1.3)] and phosphatidylcholine (Ptd, 2.78) (Table 2). Therefore, HDL, UFA, VLDL, and Ptd may be potential biomarkers of AP.

**Table 2:** Levels of macromolecular lipid metabolites in the AP and SO groups

**Chemical shift (ppm)**	**Name of compound**	**Acute pancreatitis group (AP)**
**0.86**	**HDL**	↑
**1.22**	**LDL**	**-**/↓
**1.3**	**VLDL/LDL**	↓
**0.9**	**HDL/LDL**	↓
**1.34**	**VLDL**	↓
**2.78**	**UFA**	↑
**3.22**	**Ptd**	↓
**5.3**	**UFA**	↑

**↑**: elevated; **↓**: reduced; −: similar; HDL: high density lipoprotein; LDL: low density lipoprotein; VLDL: very low density lipoprotein; FA: saturated fatty acid; Ptd: phosphatidylcholine; UFA: unsaturated fatty acid. All of the results were compared with the sham operation (SO) group.

## Discussion

By analyzing the plasma metabolites of rats with experimental AP using the 1H-NMR-based metabolomic approach, we obtained a series of finger-prints containing information on plasma metabolites. Except for one point, the separation between the two groups was distinct, as shown in the score plot of PCA models of the CPMG data for small molecules. Similarly, we observed a separation of metabolites between the two groups in the score plot of PLS-DA models for macromolecules, although the two groups were mixed by PCA.

The plasma 1H-NMR spectra of CPMG data for small molecules showed a difference between the two groups. The score plot with PCA of 1H-NMR (CPMG) spectra of plasma samples showed that most of the metabolites in the AP group could be separated from those in the SO group, indicating a significant difference in metabolomics between AP and control rats. The score plot with PCA of 1H-NMR (LED) spectra from the two groups showed a cross distribution. The cluster distribution of the PLS-DA could distinguish between the two groups, thus illustrating their metabolomic differences.

This study also showed differences in plasma metabolite levels between the two groups. Rats with AP had higher levels of lactate, valine, succinic acid, 3-hydroxybutyrate (3-HB), HDL, and UFA, and lower levels of glycerol, choline, TMAO, glucose, glycine, VLDL, and phosphatidylcholine, than the control group.

Because of acute systemic inflammation response syndrome, AP always triggers a hypercatabolic state, resulting in increased energy requirements and protein catabolism [23]. Our results indicate that multiple metabolic pathways were mobilized, resulting in changes in metabolite concentrations. Lactate is mainly generated by anaerobic glycolysis and is not associated with the primary energy supply pathway under normal conditions. During an intense workout or under conditions of hypoxia, an insufficient oxygen supply can reduce Krebs cycle reactions, produce less ATP, and enhance glycolysis, resulting in decreased removal ability of the liver and kidneys and ultimately to the accumulation of lactate in plasma [24,25]. The increased levels of lactate and succinic acid in the AP group were likely because of anaerobic glycolysis, which was enhanced by the increased energy consumption and oxygen deficit during early stages of AP [26]. Since lactate concentration is influenced by oxygen supply and fluid resuscitation, this elevation was not detected in urine samples from patients with pancreatitis [27]. As an aerobic process, gluconeogenesis was inhibited by an inadequate oxygen supply, which may be partly responsible for the decrease in glucose level. Further, insulin released from injured acinar cells also contributed to decreased glucose levels. TMAO, the product of dietary choline and L-carnitine [28] , although mainly regarded as a biomarker of renal function [29], may participate in energy production as an external electron acceptor in an hypoxic environment[30]. Moreover, decreased TMAO has been reported to accompany a chemiosmotic mechanism of energy conversion [31]. The decreased TMAO we observed may indicate renal damage caused by AP as well as energy conversion under conditions of relative oxygen deficit. Similar changes in TMAO have been observed in serum samples of patients with pancreatitis [32]. In contrast, 3-HB, a ketone body synthesized in the liver and used as an energy source by the brain under conditions of hypoglycemia or enhanced demand for gluconeogenesis [33], was not increased in serum samples of patients with pancreatitis [32]. Inflammatory responses in AP progressively damage pancreatic acinar cells and impair mitochondrial respiratory capacity, limiting ATP production [34]. In addition, increased energy consumption mobilizes FA to the periphery as an energy supply, reducing FA and increasing 3-HB. This results in increased HDL level at the periphery, where it is required to carry cholesterol and triacylglycerol. Serum VLDL transports triacylglycerol into tissue for energy metabolism, and phosphatidylcholine moves into tissue to preserve cell function, leading to elevated serum HDL levels and decreased LDL, VLDL, and phosphatidylcholine levels. UFA, which is important in maintaining the relative liquidity of cell membranes and normal cell function, is released into circulation under stress, damaging the stability of cell membranes [35]. Moreover, glutaminate production is increased to protect against tissue injury, and alanine accumulates to inhibit the proteolysis of skeletal muscle proteins, resulting in increased serum levels of glutaminate and alanine.

One of the limitations of this study was that we only investigated changes in plasma concentrations of metabolic factors. Further studies investigating urine and pancreatic tissue samples are needed to confirm our findings. Moreover, insufficient plasma was available for assay of all factors, and this study was based on the 1HNMR approach, which in itself is limited by the incomplete data obtained. The combined use of LC-MS or GC-MS is needed to validate our results.

## Conclusion

We found that the characteristic metabolomic profile differed in AP and SO rats. Lactate, valine, succinic acid, 3-HB, HDL, UFA, glycerol, choline, TMAO, glucose, glycine, VLDL, and Ptd may be regarded as potential biomarkers of early stage AP. These findings may provide deeper insight into the pathophysiology and metabolic state of AP and may facilitate early intervention in patients with this disease. The 1HNMR-based metabolomic approach was capable of distinguishing between plasma samples of AP rats and SO controls, providing a new and non-invasive methodology for the study of AP.

## Abbreviations

AP: Acute pancreatitis; CPMG: Carr-Purcell-Meiboom-Gill; DA: Discriminant analysis; FID: Free induction decays; LED: Longitudinal Eddy-delay; NMR: Nuclear magnetic resonance; OSC: Orthogonal signal correction; PLS: Partial least squares; PCA: Principal components analysis; SIMCA-P: Soft independent modeling of class analogy; LC-MS: Liquid chromatography-mass spectrometry; GC-MS: Gas chromatography–mass spectrometry; HDL/LDL/VLDL: High/low/very low density lipoprotein; FA/UFA: Saturated/unsaturated fatty acid; TMAO: Trimethylamine N-oxide; NAC: N-acetyl glycoprotein.

## Competing interests

The authors declare that they have no competing interests.

## Authors’ contributions

WFT, study concept and design; YXL, XHP and MHW, acquisition of data; analysis and interpretation of data; JL and XLZ, drafting of the manuscript; WFT, critical revision of the manuscript for important intellectual content; YXL and SFZ, statistical analysis; WFT, obtained funding; YLL and HG, material support; WFT, study supervision. All authors read and approved the final manuscript.

## Pre-publication history

The pre-publication history for this paper can be accessed here:

http://www.biomedcentral.com/1471-230X/14/115/prepub
